# Supplementary material for: Antagonizing cholecystokinin A receptor in the lung attenuates obesity-induced airway hyperresponsiveness
Source: Nat Commun. 2023 Jan 4;14:47. doi: 10.1038/s41467-022-35739-8 (PMC9813361; doi:10.1038/s41467-022-35739-8)
Supplement: Supplementary file 1 — Supplementary Information [file 41467_2022_35739_MOESM1_ESM.pdf]

Antagonizing cholecystokinin A receptor in the lung attenuates obesity-induced airway hyperresponsiveness

Supplementary Information

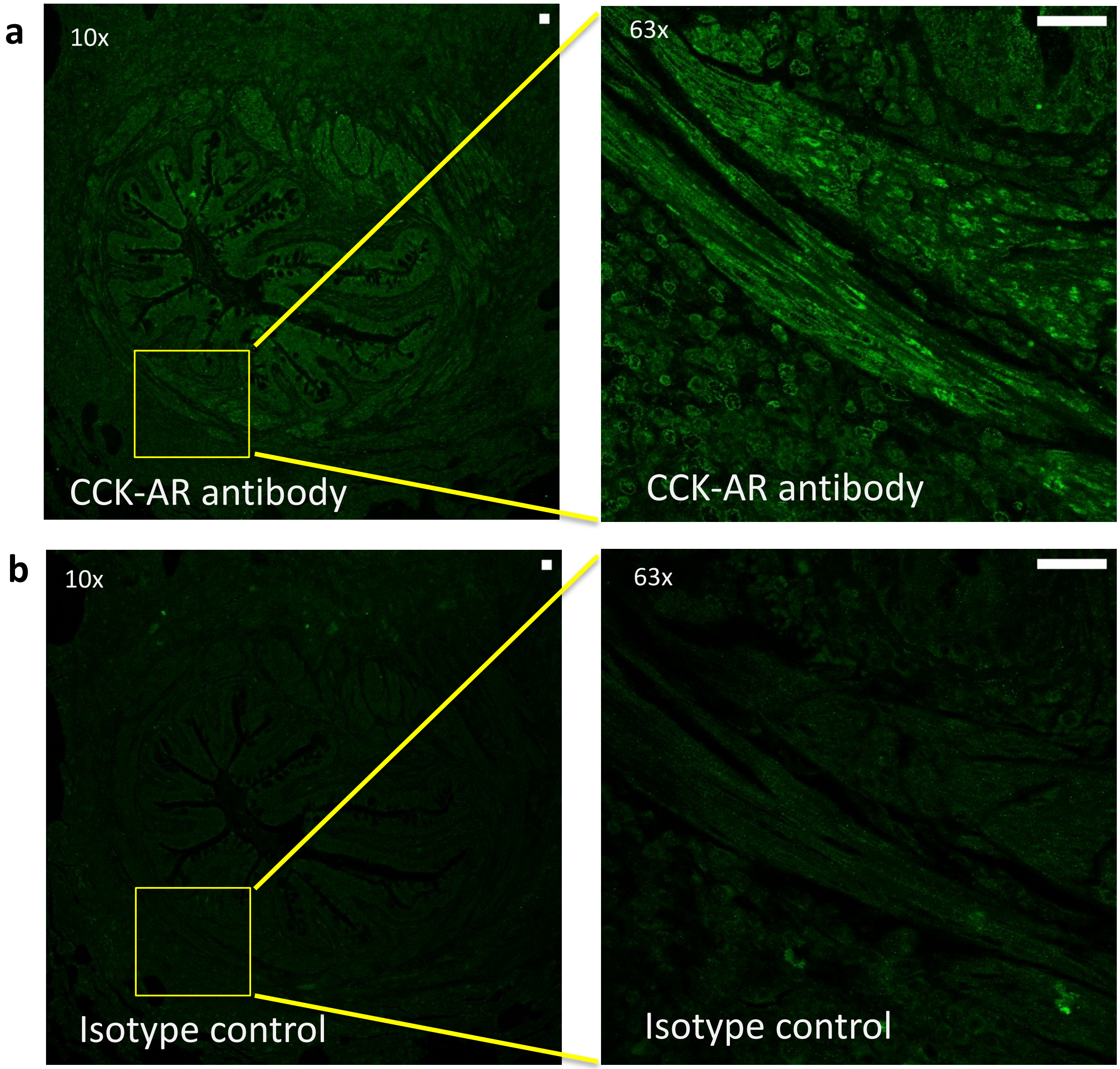

Figure S1. **Immunofluorescence staining of CCKAR in lung tissue section.** (a-b) Immunofluorescence staining of CCKAR (a) in comparison to corresponding isotype control (b) in human lung tissue section. Scale bar = 25 µm. Left panel: 10x magnification. Right panel: 63x, oil.

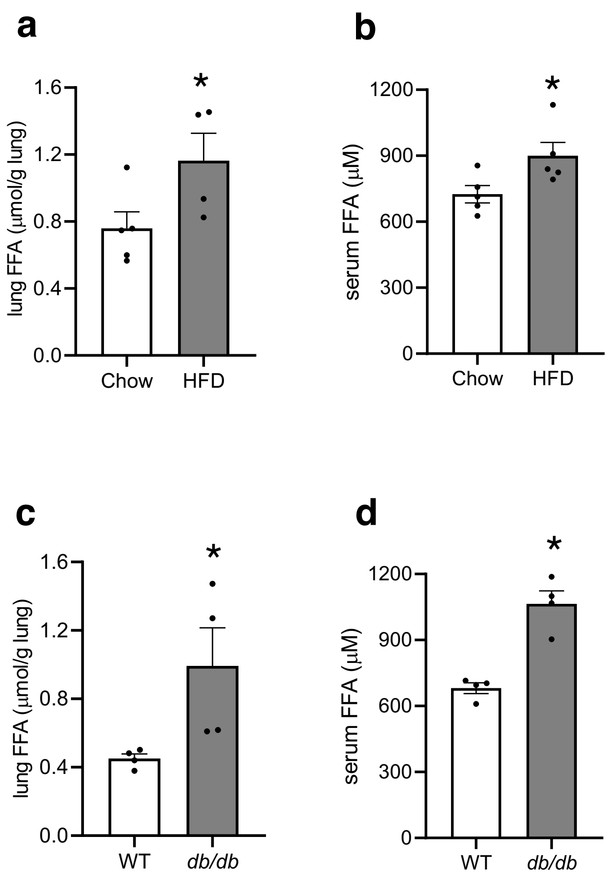

Figure S2. **Quantification of free fatty acids in obese mice versus lean controls.** a. Free fatty acid levels in the lung of HFD-fed (n=4) and regular chow-fed mice (n=5); P=0.0317. b. Free fatty acid levels in the serum of HFD-fed (n=5) and regular chow-fed mice (n=5); P=0.0217. c. Free fatty acid levels in the lung of *db/db* (n=4) and WT mice (n=4); P=0.0260. . Free fatty acid levels in the lung of *db/db* (n=4) and WT mice (n=4); P=0.0005. Data are mean +/- SEM; one-tailed, unpaired t-test (*P<0.05).

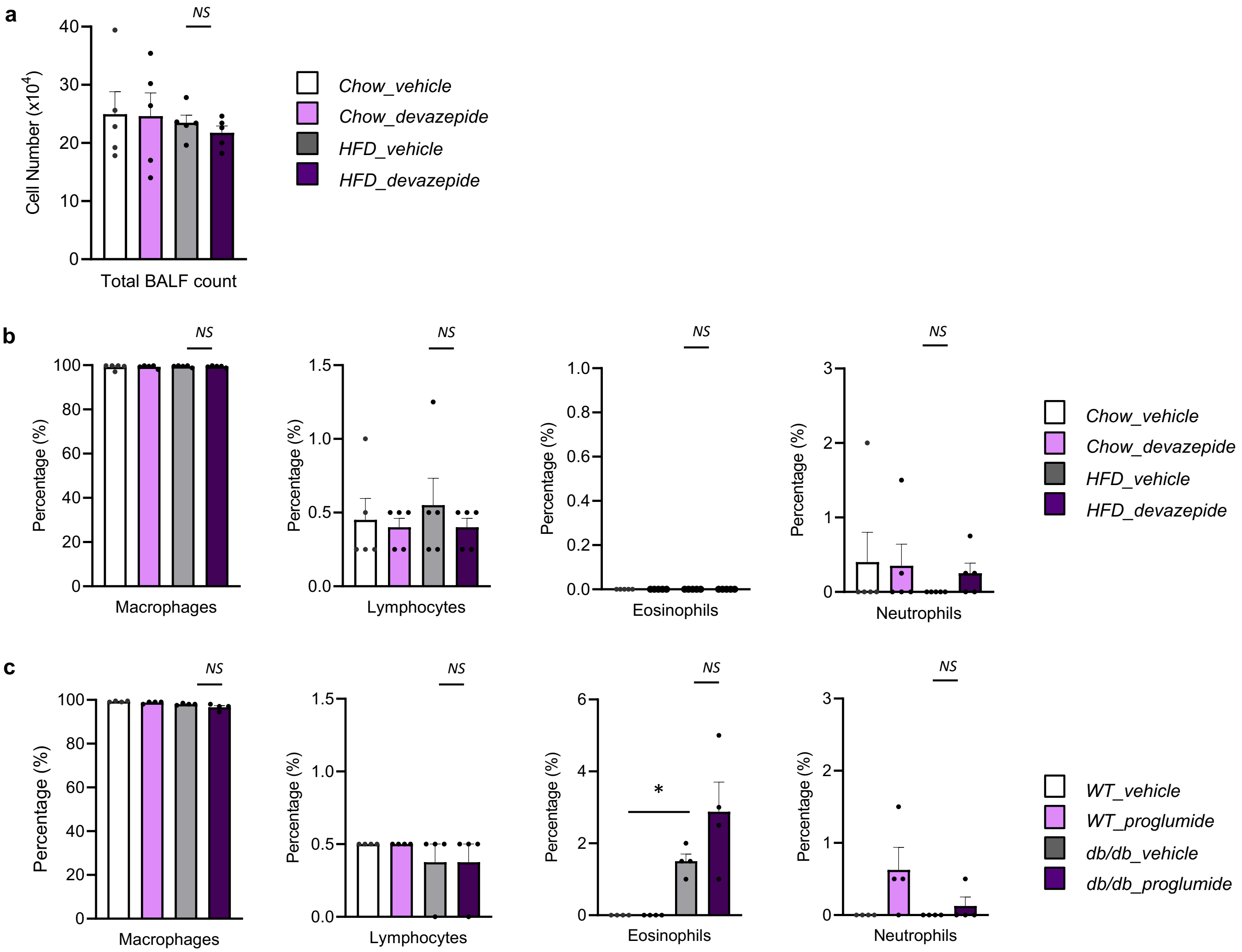

Figure S3. **BAL cell analysis in CCKAR antagonists-treated mice.** (a) Total BAL cell counts in HFD-fed mice treated with either devazepide (n=5) or vehicle (n=5) and regular chow-fed mice treated with either devazepide (n=5) or vehicle (n=5). (b) Differential cell counts in HFD-fed mice treated with either devazepide (n=5) or vehicle (n=5) and regular chow-fed mice treated with either devazepide (n=5) or vehicle (n=5). (b) Differential cell counts in *db/db* mice treated with either proglumide (n=4) or vehicle (n=4) and wild type mice treated with either proglumide (n=4) or vehicle (n=4). P=0.0002 for *db/db*-vehicle eosinophil group vs wild type-vehicle eosinophils group. Data are mean +/- SEM; one-tailed, unpaired t-test (*P<0.05). *NS*=not significant.

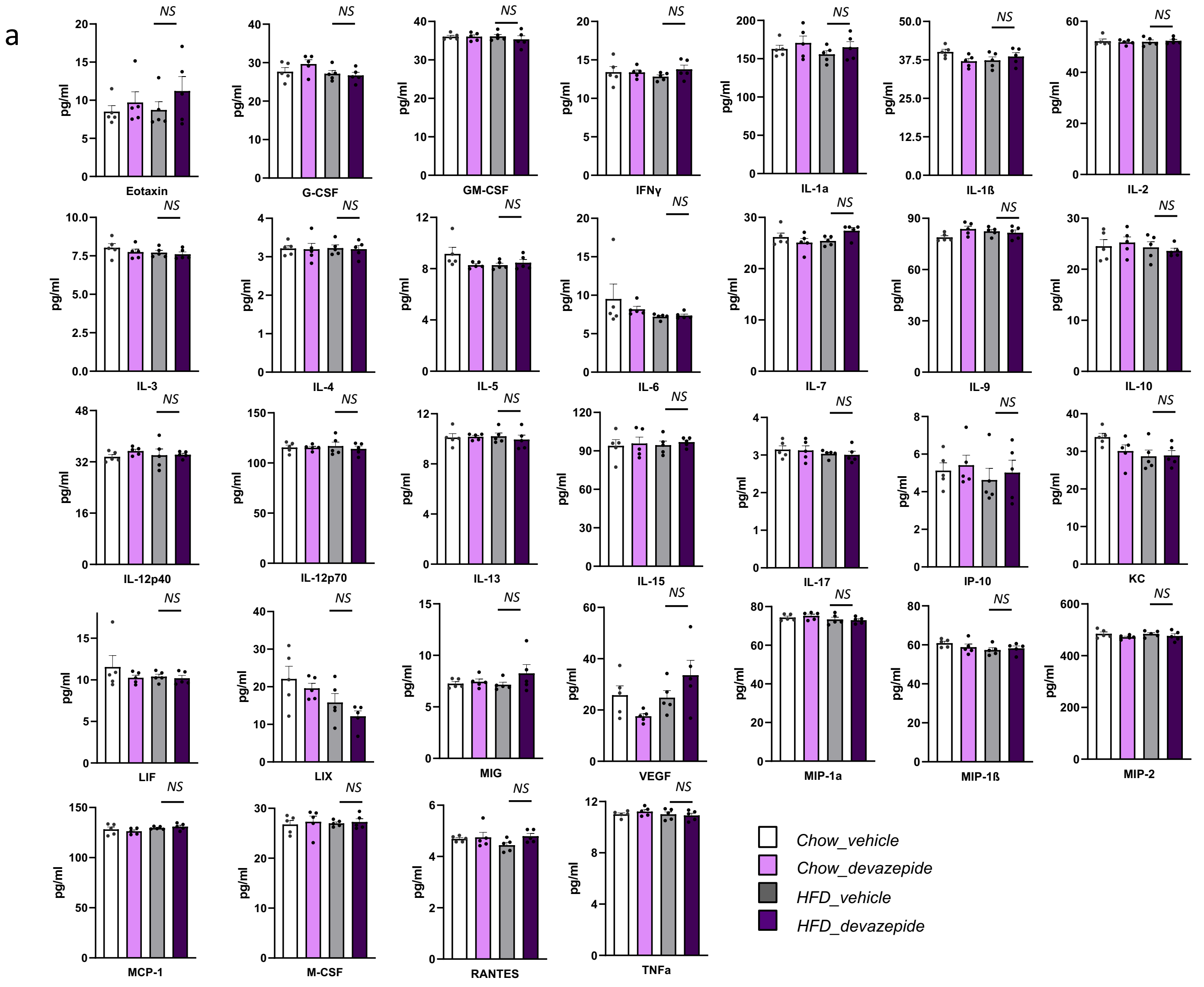

Figure S4**. Levels of cytokines in BAL of CCKAR antagonists-treated mice.** Inflammatory cytokines were assessed by multiplex cytokine assay (EVE Technologies, AB Canada) in BAL of HFD-fed mice treated with either devazepide or vehicle and regular chow-fed mice treated with either devazepide of vehicle (n=5 mice per group) (a) and *db/db* mice treated with either proglumide or vehicle and wild type mice treated with either devazepide or vehicle (n=4 mice per group) (b, figure below). Data are mean +/- SEM. one-tailed, unpaired t-test (*P<0.05). *NS*=not significant.

b

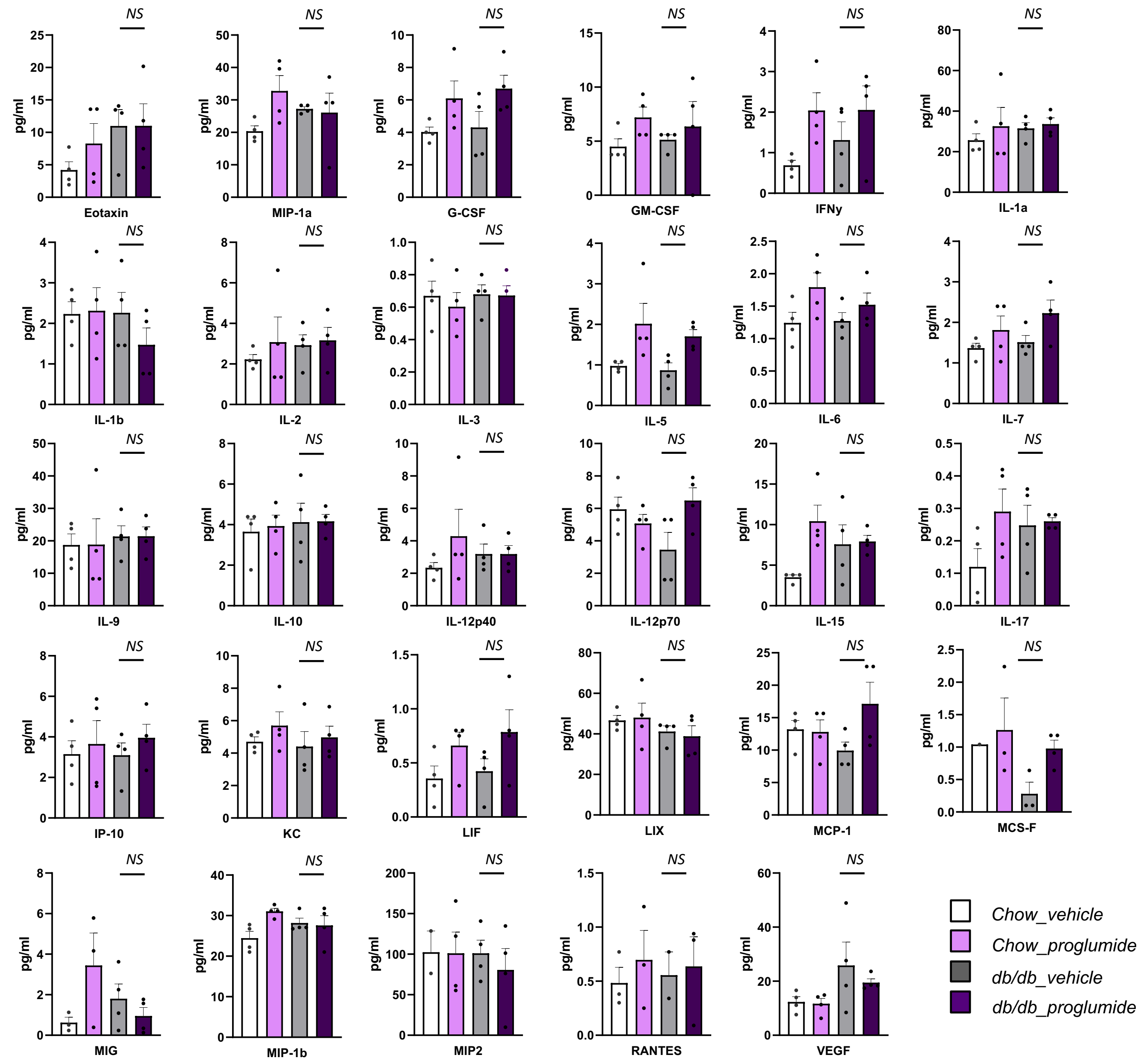

Figure S4**. Levels of cytokines in BAL of CCKAR antagonists-treated mice.** Inflammatory cytokines were assessed by multiplex cytokine assay (EVE Technologies, AB Canada) in BAL of HFD-fed mice treated with either devazepide or vehicle and regular chow-fed mice treated with either devazepide of vehicle (n=5 mice per group) (a, figure above) and *db/db* mice treated with either proglumide or vehicle and wild type mice treated with either devazepide or vehicle (n=4 mice per group) (b). Data are mean +/- SEM. one-tailed, unpaired t-test (*P<0.05). *NS*=not significant.
