# Supplementary material for: Antagonizing cholecystokinin A receptor in the lung attenuates obesity-induced airway hyperresponsiveness
Source: Nat Commun. 2023 Jan 4;14:47. doi: 10.1038/s41467-022-35739-8 (PMC9813361; doi:10.1038/s41467-022-35739-8)
Supplement: Supplementary file 3 — 'Description of Additional Supplementary Files [file 41467_2022_35739_MOESM3_ESM.docx]

**Description of Additional Supplementary Files**

File Name: Supplementary Data

Expression of non-olfactory GPCRs in primary human ASM cells. Using previously published RNA-seq dataset (available at the Gene Expression Omnibus Web site (<http://www.ncbi.nlm.nih.gov/geo/>) under accession [GSE52778](https://www.ncbi.nlm.nih.gov/geo/query/acc.cgi?acc=GSE52778)), the expression of each non-olfactory GPCRs (361 total) were assessed based on based on the values of Fragments Per Kilobase of transcript per Million mapped reads (FPKM). From this analysis, 115 (~32%) non-olfactory GPCRs that are moderately-to-highly expressed (FPKM>0.1) in ASM cells
