# Supplementary material for: Antagonizing cholecystokinin A receptor in the lung attenuates obesity-induced airway hyperresponsiveness
Source: Nat Commun. 2023 Jan 4;14:47. doi: 10.1038/s41467-022-35739-8 (PMC9813361; doi:10.1038/s41467-022-35739-8)
Supplement: Supplementary file 4 — Supplementary Data [file 41467_2022_35739_MOESM4_ESM.pdf]

tracking_id	gene_id	tss_id	locus	FPKM
BDKRB2	BDKRB2	TSS15904	chr14:96671134-9671066	91.0771
GPR124	GPR124	TSS20121	chr8:37654400-37707431	82.5985
F2R	F2R	TSS11220	chr5:76011867-76031595	74.9328
GPR133	GPR133	TSS21901	chr12:131438451-131626	53.0235
CCKAR	CCKAR	TSS15042	chr4:26483017-26492042	49.6329
GPR176	GPR176	TSS2714	chr15:40092930-4021309	46.7417
CCRL1	CCRL1	TSS20357,TSS24985	chr3:132276981-1325930	41.992
CXCR7	CXCR7	TSS27807	chr2:237478379-2374909	33.3002
F2RL2	F2RL2	TSS13749,TSS6218	chr5:75699148-76003957	32.6355
GLIPR2	GLIPR2	TSS27557	chr9:36136741-36163903	32.02
CHRM2	CHRM2	TSS16537,TSS4231,T	chr7:136553398-1368490	29.7128
GLIPR1	GLIPR1	TSS22611	chr12:75874512-7590541	25.8561
GPR108	GPR108	TSS21643	chr19:6729924-6737633	25.2228
FZD7	FZD7	TSS13101	chr2:202899309-2029031	23.8696
FZD2	FZD2	TSS19747	chr17:42634811-4263863	20.2433
FZD1	FZD1	TSS27526	chr7:90893782-90898132	19.7195
GPR107	GPR107	TSS25646	chr9:132815984-1329024	18.5615
PTGFR	PTGFR	TSS23248	chr1:78956727-79006386	17.3505
GPRC5B	GPRC5B	TSS7004	chr16:19870292-1989615	14.751
GPR137	GPR137	TSS15587,TSS17726,	chr11:64037299-6405697	13.7498
BDKRB1	BDKRB1	TSS21118	chr14:96722546-9673110	13.2884
GPR172A	GPR172A	TSS21548,TSS28050,	chr8:145582216-1455849	12.6164
TM7SF3	TM7SF3	TSS12027	chr12:27124505-2716733	11.9126
GPR137B	GPR137B	TSS10438	chr1:236305831-2363722	11.8705
FZD6	FZD6	TSS20186,TSS22367	chr8:104310660-1043450	10.0788
GPR153	GPR153	TSS7510	chr1:6307405-6321035	9.6119
FZD4	FZD4	TSS23321	chr11:86656716-8666644	9.29699
HRH1	HRH1	TSS19052,TSS24303,	chr3:11178778-11304939	8.43063
P2RX4	P2RX4	TSS9268	chr12:121647663-121671	8.40913
GPR161	GPR161	TSS16430	chr1:168053996-1681056	8.3355
CD97	CD97	TSS6578	chr19:14491955-1451953	8.14483
GPR125	GPR125	TSS18787	chr4:22388998-22517672	6.64568
GPRC5A	GPRC5A	TSS10189	chr12:13043955-1306660	6.0685
GPR1	GPR1	TSS6123	chr2:207040041-2070827	5.71962
GABBR1	GABBR1	TSS11540,TSS13357,	chr6:29570004-29600962	5.26914
RAI1	RAI1	TSS19609	chr17:17584786-1774032	5.07959
BAI2	BAI2	TSS17450	chr1:32192717-32229648	5.01084
PTGER4	PTGER4	TSS7272	chr5:40680031-40693837	4.85337
OPN3	OPN3	TSS162	chr1:241695433-2418037	4.8463
SMO	SMO	TSS5402	chr7:128828712-1288533	4.73042
GPR89A	GPR89A	TSS26883	chr1:145764594-1458271	4.65535
PTGER2	PTGER2	TSS15417	chr14:52781015-5279532	4.63789
EDNRB	EDNRB	TSS12723,TSS24089,	chr13:78469615-7854966	4.63395
LGR4	LGR4	TSS1265	chr11:27387507-2749433	4.19559
GPR89B	GPR89B	TSS12321	chr1:147400505-1474657	3.71404
GPRASP2	GPRASP2	TSS11244	chrX:101854095-1019726	3.61921

GPR126	GPR126	TSS22593	chr6:142623055-14276740	3.57682
PTGER3	PTGER3	TSS24031	chr1:71318035-71546972	3.47301
GPR89C	GPR89C	TSS1032,TSS22541	chr1:145883867-14592404	3.1011
GPR162	GPR162	TSS620	chr12:6930962-6936583	2.69106
SSTR1	SSTR1	TSS22681	chr14:38677203-38682268	2.62883
HTR7P1	HTR7P1	TSS23735	chr12:13153375-13157764	2.51078
ADORA2B	ADORA2B	TSS21991	chr17:15848230-15879210	2.48436
OXTR	OXTR	TSS18517	chr3:8792094-8811300	2.28825
GPR75-ASB3	GPR75-ASB3	TSS22265	chr2:53897116-54087170	2.16848
MC1R	MC1R	TSS19727	chr16:89984286-89987385	2.16023
P2RX7	P2RX7	TSS20339,TSS27017	chr12:121570621-1216243	2.04314
EDNRA	EDNRA	TSS1245	chr4:148402068-14846610	1.92455
P2RY1	P2RY1	TSS11305	chr3:152552735-15255584	1.83687
PTGIR	PTGIR	TSS19026	chr19:47123724-47128354	1.78658
GPRASP1	GPRASP1	TSS10808,TSS7947	chrX:101854095-10197260	1.75512
GPR37	GPR37	TSS25665	chr7:124386113-12440568	1.6224
GPR68	GPR68	TSS25108,TSS8700	chr14:91698875-91720224	1.56938
TBXA2R	TBXA2R	TSS26976	chr19:3594503-3606831	1.48326
CCR10	CCR10	TSS25094	chr17:40831419-40833845	1.38977
AGTR1	AGTR1	TSS10496,TSS19229	chr3:148415657-14846079	1.37023
GPR173	GPR173	TSS7210	chrX:53078505-53109796	1.29994
P2RX6	P2RX6	TSS15680,TSS8148	chr22:21369441-21382303	1.28335
GPR155	GPR155	TSS22	chr2:175296374-1753518	1.2397
PTH1R	PTH1R	TSS22968,TSS23466	chr3:46919235-46945289	1.23173
GPR180	GPR180	TSS17611	chr13:95254103-95286899	1.21939
ADORA1	ADORA1	TSS7660	chr1:203096835-20313653	1.15116
OPRL1	OPRL1	TSS146,TSS18922	chr20:62711470-62731990	1.07569
CCRN4L	CCRN4L	TSS27899	chr4:139936942-13996709	1.04152
GPR135	GPR135	TSS6004	chr14:59930239-59932059	0.844058
ADRA2A	ADRA2A	TSS25014	chr10:112836789-1128400	0.842078
GPR56	GPR56	TSS16552,TSS25502,	chr16:57653909-57698944	0.723415
GABBR2	GABBR2	TSS11675	chr9:101050363-10147147	0.713211
F2RL1	F2RL1	TSS27007	chr5:76114832-76131140	0.711562
GPRC5C	GPRC5C	TSS28191,TSS6407	chr17:72427666-72443568	0.661979
ADRA1B	ADRA1B	TSS18546	chr5:159343739-1594000	0.651025
LTB4R	LTB4R	TSS26864,TSS3948	chr14:24774392-24787243	0.62234
FZD8	FZD8	TSS13759	chr10:35927176-35930362	0.597953
GPR146	GPR146	TSS18801	chr7:1036622-1177893	0.578975
P2RY11	P2RY11	TSS1247	chr19:10216898-10230599	0.540341
GPR157	GPR157	TSS6057	chr1:9164475-9189229	0.526146
HTR2B	HTR2B	TSS5255	chr2:231921577-23203754	0.484629
HTR2B	HTR2B	TSS5255	chr2:231921577-23203754	0.484629
GPR4	GPR4	TSS15128	chr19:46093022-46105460	0.439498
ADRA1D	ADRA1D	TSS9239	chr20:4201277-4229659	0.436219
CCR7	CCR7	TSS22025	chr17:38710021-38721730	0.428315
GPR160	GPR160	TSS7866	chr3:169755734-16980318	0.422801
GPR137C	GPR137C	TSS16230	chr14:53019865-5310443	0.395529
PTGDR2	PTGDR2	TSS18528	chr11:60609428-60623444	0.349219

PTGER1	PTGER1	TSS23015	chr19:14583277-1458617	0.340782
CALCRL	CALCRL	TSS17119	chr2:188207848-1883130	0.31648
GPR85	GPR85	TSS10255,TSS1174,T	chr7:112720467-1127278	0.302688
C5AR1	C5AR1	TSS18038	chr19:47813103-4782532	0.293195
GPR35	GPR35	TSS11312,TSS12014	chr2:241544824-2415706	0.286359
CELSR3	CELSR3	TSS4478	chr3:48673895-48700348	0.283246
ADRB2	ADRB2	TSS1360	chr5:148206155-1482081	0.27282
LTB4R2	LTB4R2	TSS242	chr14:24774392-2478724	0.263722
P2RX5	P2RX5	TSS1330	chr17:3539761-3599698	0.259111
CELSR2	CELSR2	TSS27190	chr1:109792640-1098183	0.25551
GPR75	GPR75	TSS22265	chr2:53897116-54087170	0.254858
GPR39	GPR39	TSS16990	chr2:133174146-1334290	0.249934
GPR63	GPR63	TSS11869	chr6:97241997-97285353	0.219425
GPR17	GPR17	TSS13902	chr2:128395995-1284393	0.188669
P2RY14	P2RY14	TSS16142,TSS20981	chr3:150804675-1511764	0.188383
FZD9	FZD9	TSS25043	chr7:72848108-72850450	0.182975
GLIPR1L2	GLIPR1L2	TSS3716	chr12:75784888-7581782	0.178153
DRD4	DRD4	TSS21500	chr11:637304-640705	0.139598
ADORA2A	ADORA2A	TSS26182	chr22:24823529-2489078	0.138636
CCBP2	CCBP2	TSS26978	chr3:42850963-42908775	0.129838
FZD5	FZD5	TSS23229	chr2:208627309-2086341	0.108118
