# Supplementary material for: Antagonizing cholecystokinin A receptor in the lung attenuates obesity-induced airway hyperresponsiveness
Source: Nat Commun. 2023 Jan 4;14:47. doi: 10.1038/s41467-022-35739-8 (PMC9813361; doi:10.1038/s41467-022-35739-8)
Supplement: Supplementary file 5 — Reporting Summary [file 41467_2022_35739_MOESM5_ESM.pdf]

nature research

Corresponding author(s): Quan Lu

Last updated by author(s): 17/12/2022

Reporting Summary

Nature Research wishes to improve the reproducibility of the work that we publish. This form provides structure for consistency and transparency in reporting. For further information on Nature Research policies, see Authors & Referees and the Editorial Policy Checklist .

Statistics

For all statistical analyses, confirm that the following items are present in the figure legend, table legend, main text, or Methods section.

n/a	Confirmed	
☐	☒	The exact sample size (*n*) for each experimental group/condition, given as a discrete number and unit of measurement
☐	☒	A statement on whether measurements were taken from distinct samples or whether the same sample was measured repeatedly
☐	☒	The statistical test(s) used AND whether they are one- or two-sided<br>*Only common tests should be described solely by name; describe more complex techniques in the Methods section.*
☒	☐	A description of all covariates tested
☐	☒	A description of any assumptions or corrections, such as tests of normality and adjustment for multiple comparisons
☐	☒	A full description of the statistical parameters including central tendency (e.g. means) or other basic estimates (e.g. regression coefficient) AND variation (e.g. standard deviation) or associated estimates of uncertainty (e.g. confidence intervals)
☐	☒	For null hypothesis testing, the test statistic (e.g. *F*, *t*, *r*) with confidence intervals, effect sizes, degrees of freedom and *P* value noted<br>*Give P values as exact values whenever suitable.*
☒	☐	For Bayesian analysis, information on the choice of priors and Markov chain Monte Carlo settings
☒	☐	For hierarchical and complex designs, identification of the appropriate level for tests and full reporting of outcomes
☒	☐	Estimates of effect sizes (e.g. Cohen's *d*, Pearson's *r*), indicating how they were calculated

Our web collection on statistics for biologists contains articles on many of the points above.

Software and code

Policy information about availability of computer code

Data collection	For Data Collection:<br>Step One Plus Real-time PCR System<br>SpectraMax 190 Microplate Reader, Molecular Devices;<br>SpectraMax 190 Microplate Reader, Molecular Devices<br>Leica SP8X Confocal; Microscope<br>flexiVent (flexiWare7 version, Scireq)
Data analysis	For Statistical analysis (Prism v8.2.1; GraphPad Software Inc. for Statistical analysis)<br>For Image processing (ImageJ2 ImageJ2<br>Version: 2.3.0/1.53q<br>Build:d544a3f481)

For manuscripts utilizing custom algorithms or software that are central to the research but not yet described in published literature, software must be made available to editors/reviewers. We strongly encourage code deposition in a community repository (e.g. GitHub). See the Nature Research guidelines for submitting code & software for further information.

Data

Policy information about availability of data

All manuscripts must include a data availability statement. This statement should provide the following information, where applicable:

- Accession codes, unique identifiers, or web links for publicly available datasets
- A list of figures that have associated raw data
- A description of any restrictions on data availability

The data in Fig. 1a and Supplementary Table were generated from a previously published RNA-seq data available at the Gene Expression Omnibus Web site (http://www.ncbi.nlm.nih.gov/geo/) under accession GSE52778. All data will be made available from the corresponding author upon request.

Field-specific reporting

Please select the one below that is the best fit for your research. If you are not sure, read the appropriate sections before making your selection.

☒ Life sciences ☐ Behavioural & social sciences ☐ Ecological, evolutionary & environmental sciences

For a reference copy of the document with all sections, see nature.com/documents/nr-reporting-summary-flat.pdf

Life sciences study design

All studies must disclose on these points even when the disclosure is negative.

Sample size	For flexivent experiments, at least 5 mice were used for each treatment group. This number of mice is sufficient to allow us to perform robust statistics on AHR and other phenotypes among different treatment/interventions based on previous studies. For other mouse experiments, at least 4 animals were used. Number of animals used in the experiments are noted in the legends.<br>For cell culture experiments, no sample size calculation was performed. At least 3 independent samples per group were used which is generally sufficient to perform statistics by t-test
Data exclusions	No experimental data were intentionally excluded. For lung mechanics/flexivent experiments, data were excluded for subjects that died during/after the flexivent (e.g. accidental trachea and lung perforation, but not because of antagonist treatment); this is known to alter the response to methacholine and is generally excluded in the analysis .
Replication	Triplicates were included for most cell-based assays. 5-6 mice were used for each experimental condition. For the lung mechanics/flexivent experiments, different models (db/db and high fat diet-fed mice) and different antagonists (proglumide and devazepide) were used. For cell culture experiments, different antagonists (proglumide, lorglumide, devazepide) as well as genetic ablation were used. For experiments that were repeated for replication, similar results were obtained.
Randomization	For mouse experiments, mice were randomly assigned to the different treatment groups. Similarly, for cell culture experiments, samples were randomly assigned into experimental groups
Blinding	No blinding was conducted for most experiments where the scientist that performed the treatment was also the one that conducted the assays. In cases were at least two scientists were involved, whenever possible, one was blinded of the treatment groups. Whenever possible, for both cell culture and mouse experiments, the person who performed the analysis was different from the one who performed the experiment. For BAL measurements conducted by a commercial company (e.g. EVE), samples sent were only labeled alpha-numerically without description.

Reporting for specific materials, systems and methods

We require information from authors about some types of materials, experimental systems and methods used in many studies. Here, indicate whether each material, system or method listed is relevant to your study. If you are not sure if a list item applies to your research, read the appropriate section before selecting a response.

Materials & experimental systems

n/a	Involved in the study	
☐	☒	Antibodies
☐	☒	Eukaryotic cell lines
☒	☐	Palaeontology
☐	☒	Animals and other organisms
☒	☐	Human research participants
☒	☐	Clinical data

Methods

n/a	Involved in the study	
☒	☐	ChIP-seq
☒	☐	Flow cytometry
☒	☐	MRI-based neuroimaging

Antibodies

Antibodies used	Primary antibodies for immunostaining used were anti-CCKAR (Pierce, Cat. PA3-116), anti-CCK (Santa Cruz Biotechnology, Inc., Cat. Sc-21617), and anti-alpla-SMA (Sigma, Cat. C6198).
Validation	PA3-116 has been successfully used in Immunocytochemistry and immunohistochemistry procedures as reported by the company's website. Sc-21617 was used to detect CCK expression in mouse brain and mouse hypothalamus extracts as described in the antibody datasheet; select product citations were also included in the datasheet provided by the company. C6198 is an anti-alpha-SMA mouse monoclonal antibody (Clone 1A4) purified from hybridoma cell culture. C6198 antibody reacts with normal and neoplastic, human vascular and visceral, smooth muscle cells and cross reacts with with actin in human and mouse tissues as reported by provided datasheet.

Eukaryotic cell lines

Policy information about cell lines

Cell line source(s)	Primary cultures of human ASM cells were provided by Dr. Reynold Panettieri (Rutgers University); Normal human bronchial epithelial (HBE) cells were obtained from Lonza (Cat. CC-2541). HEK293T cells were obtained from ATCC (Cat CRL-3216).
Authentication	Cells were authenticated by specific cell markers. Cells of lower passages 3-8 were used
Mycoplasma contamination	Cultured cells were regularly checked for Mycoplasma contamination. Only Mycoplasma-negative cells were used for experiments.
Commonly misidentified lines (See ICLAC register)	*Name any commonly misidentified cell lines used in the study and provide a rationale for their use.*

Animals and other organisms

Policy information about studies involving animals; ARRIVE guidelines recommended for reporting animal research

Laboratory animals	Mice were purchased from The Jackson Laboratory (Bar Harbor, Maine). Db/db mice and their WT controls were either male or female (10-12 weeks of age) while HFD-fed mice and regular chow-fed mice were 21-24 weeks of age and are only male. Strains of mice used were: C57C57BL/6J and (B6.BKS(D)-Leprdb/J).<br>A maximum of 4 mice per cage were permitted and animals were checked at least weekly after arrival. Standard monitoring practices were applied for animals including monitoring for persistent recumbence, intractable pain, severe weight loss, tumor, severe central nervous system signs, dyspnea and cyanosis, prolapse of the penis or rectum, limb or spinal fractures, and dystocia. Water and enrichment (nestlet) were also provided in the cages (static caging system). Temperature in the animal room was maintained at 70 F and relative humidity at 50%. Light and dark cycle was from 7:00 am to 7:00 pm.
Wild animals	The study did not involve wild animals.
Field-collected samples	The study did not involve samples collected from the field.
Ethics oversight	The animal experiments were approved by the Harvard Medical Area Institutional Animal Care and Use Committee (HMA-IACUC) under the protocol #IS506-6

Note that full information on the approval of the study protocol must also be provided in the manuscript.
